# Epigenomic Modifications Mediating Antibody Maturation

**DOI:** 10.3389/fimmu.2018.00355

**Published:** 2018-02-26

**Authors:** Emily C. Sheppard, Rikke Brandstrup Morrish, Michael J. Dillon, Rebecca Leyland, Richard Chahwan

**Affiliations:** ^1^Living Systems Institute, University of Exeter, Exeter, United Kingdom; ^2^Sheffield Hallam University, Sheffield, United Kingdom

**Keywords:** epigenetic modifications, epigenomics and epigenetics, antibody diversity, cytosine deamination, somatic hypermutation, class-switch recombination, B cell maturation

## Abstract

Epigenetic modifications, such as histone modifications, DNA methylation status, and non-coding RNAs (ncRNA), all contribute to antibody maturation during somatic hypermutation (SHM) and class-switch recombination (CSR). Histone modifications alter the chromatin landscape and, together with DNA primary and tertiary structures, they help recruit Activation-Induced Cytidine Deaminase (AID) to the immunoglobulin (Ig) locus. AID is a potent DNA mutator, which catalyzes cytosine-to-uracil deamination on single-stranded DNA to create U:G mismatches. It has been shown that alternate chromatin modifications, in concert with ncRNAs and potentially DNA methylation, regulate AID recruitment and stabilize DNA repair factors. We, hereby, assess the combination of these distinct modifications and discuss how they contribute to initiating differential DNA repair pathways at the Ig locus, which ultimately leads to enhanced antibody–antigen binding affinity (SHM) or antibody isotype switching (CSR). We will also highlight how misregulation of epigenomic regulation during DNA repair can compromise antibody development and lead to a number of immunological syndromes and cancer.

## Chromatin Landscape Modulates DNA Repair and Antibody Diversification

B cells experience dramatic fluctuations in their epigenomic landscape throughout hematopoiesis. During B cell development, the genetic rearrangement of germline variable (V), diversity (D), and joining (J) gene segments in the Immunoglobulin (Ig) heavy-chain locus (Igh) and V and J gene segments in the Ig light chain locus (Igk) creates a diverse B-cell receptor (BCR) repertoire, which mediates a primary antibody response upon antigen encounter. To ensure an effective and long-lasting antibody response upon binding of antigen to the BCR, in a T-cell dependent response, B-cells are triggered to enter the germinal center (GC) microenvironment. Here, the affinity of the BCR is increased *via* a process called somatic hypermutation (SHM) and the class of the constant region is switched to increase the effector function in a process called class-switch recombination (CSR). Subsequently, class switched B-cells expressing a high-affinity BCR will be positively selected in the light zone of the GC and will differentiate into long lived plasma cells and memory B-cells. It has become increasingly apparent that epigenetic modifications are indispensable for the antibody maturation processes during SHM and CSR at antibody producing genes. Both SHM and CSR are initiated by the mutator protein, Activation-Induced Cytidine Deaminase (AID), which catalyzes cytosine-to-uracil deaminations on single-stranded DNA (ssDNA) at Ig genes, to create U:G mismatches, which ultimately leads to immune diversity (1). It is the divergent downstream processing of this regulated DNA damage, by DNA repair mechanisms, which forms the highly mutated antibody-binding variable (V) regions in SHM. This ultimately gives rise to BCRs of differing affinities. Furthermore, the double-stranded breaks at the Switch (S) regions integral for CSR, give rise to a range of BCR constant regions which results in secretion of antibodies with varying effector functions (2, 3).

**Figure 1:**
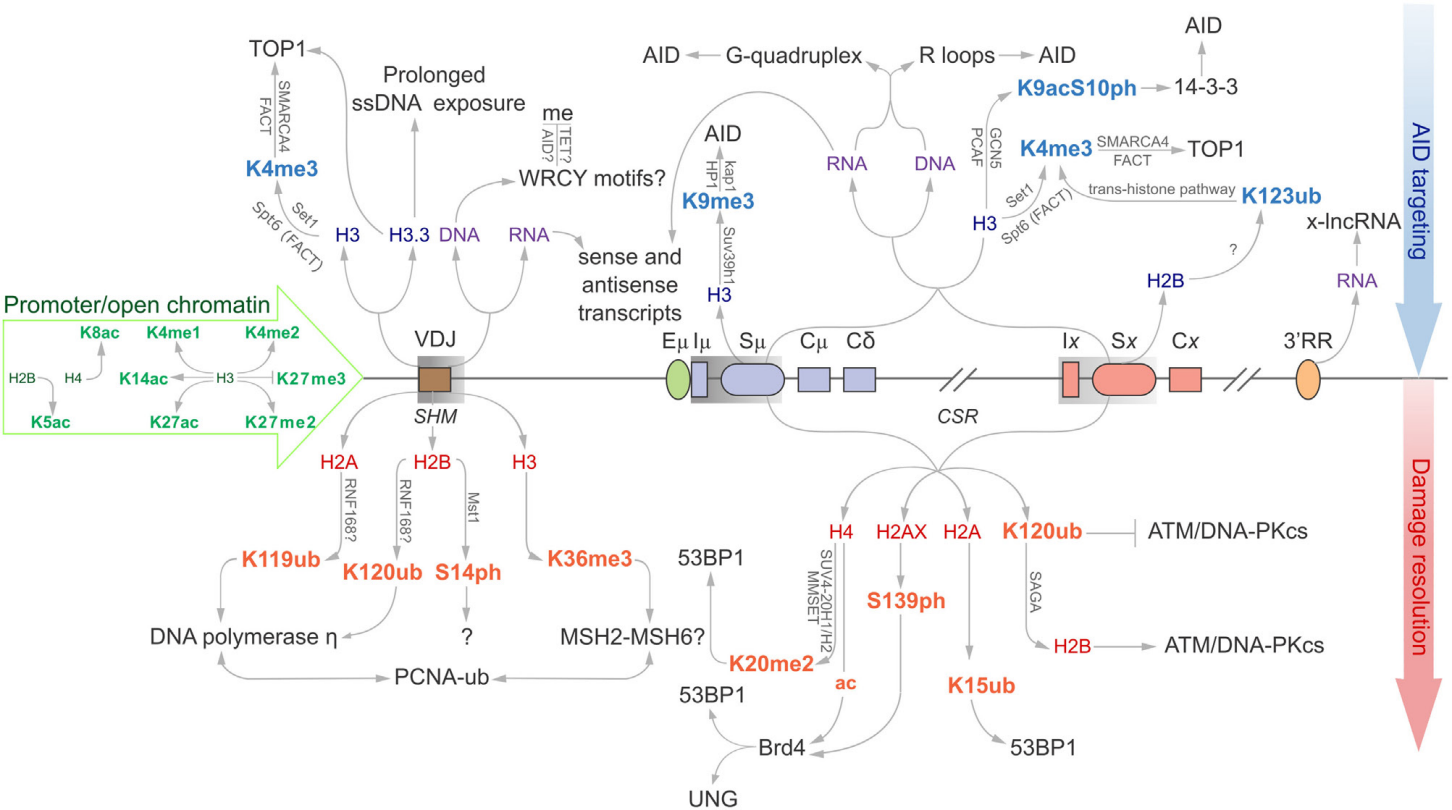
Epigenomic modifications directing antibody-diversification processes somatic hypermutation (SHM) and CSR. Green core histones and associated modifications are involved in chromatin de-compaction and enable transcription through the immunoglobulin (Ig) locus. All factors above the locus are important for the generation of DSBs while everything below encourages mutagenic repair at the V region, and DSB repair at donor and acceptor S regions (Sμ and Sx, respectively). Blue histones and affiliated modifications help recruit or tether AID and other factors that facilitate production of DSBs. Purple DNA and RNA are linked with sequences and structures that facilitate AID recruitment or targeting. Red core histones and accessory modifications recruit DNA repair proteins to ensure excision of intervening C_H_ region for successful class switching as well as error-prone polymerases to the V region.

Precursory circulating IgD^+^ naïve B cells that have yet to undergo antibody diversification have hypermethylated Ig loci and minimal histone acetylation signatures, rendering the underlying DNA inaccessible to transcriptional machinery and AID catalysis. This is in stark contrast to activated GC B cells, which accumulate open chromatin marks at the Ig loci that correlate with the induction of SHM and CSR, and the onset of transcription-coupled AID-dependent mutations (4–6). Specific histone modifications are responsible for relaxing local chromatin structure (such as H3K4me3 H3K14ac), whereas others directly propagate DNA repair pathways (such as H2AK119ub and H4K20me2; discussed below). More recently, both histone marks and RNA-based structures have been implicated in targeting AID to the Ig locus (Figure 1) (6, 7).

The physiological activity of AID is critical to maintain immune diversity, while high-fidelity DNA repair factors are important to maintain genome integrity. Misregulation of, or mutations in, these DNA repair processes can have serious consequences, spanning cancerous transformation (8), developmental defects (9), autoimmunity (10), and immunodeficiency syndromes (11). In this review, we aim to provide a cohesive understanding of higher-order epigenomic processes critical for the regulation of B cell maturation, manipulation of DNA repair mechanisms, and insights into the development of debilitating cancer- and immune-based diseases.

## Epigenomic Factors Target AID to V Regions for SHM

Somatic hypermutation enhances antibody affinity through the accumulation of point mutations at the antigen-binding V region (12). Histone marks help target AID to key sites of the Ig locus. AID preferentially deaminates cytosines in WRC motifs. These AID “hotspots” are present in Ig genes undergoing SHM (IgH, Igκ, Igλ) and CSR (IgH), which are mutated in high abundance. However, these hotspots are also prevalent at non-Ig genes, but carry significantly less mutational load (13), indicating that the presence of these hotspots alone is insufficient to recruit AID. Rather, higher-order mechanisms must be in place to regulate AID activity and targeting. RNA structures, specifically coding messenger RNAs (mRNA), non-coding RNAs (ncRNAs), and defined histone signatures, represent additional mechanisms for AID targeting.

### Role of mRNA and ncRNA in SHM

Sense mRNA transcripts have been detected at Cμ regions, which seem refractory for AID-induced mutations, while both sense and antisense transcripts have been observed at the neighboring V and S regions (7). Interestingly, V and S regions are susceptible to AID deaminations, but not C regions. Whether this is due to efficient error-free repair or lack of AID targeting remains to be addressed (14–16). The sense and antisense transcripts are thought to be free to bind to complementary regions on both stands of the transcription bubble during SHM and CSR. This forms an R-loop, a three-stranded DNA:RNA hybrid and the associated non-template ssDNA that can provide a ready target for AID. This should be reflected in the mutation profile observed at the V region, which should be equally prolific along the V region. Instead, most mutations take place within the first few hundred base pairs, before tapering off as distance from the TSS increases. Antisense transcripts originating from downstream of the recombined VDJ region should compensate for this, and AID should be equally able to access this downstream DNA. As there is no clearly defined antisense TSS, it is possible that there is reduced antisense transcription relative to sense. It is also possible that antisense transcripts suffer shorter half-lives (17). Regardless, this offers further proof that RNA transcripts support SHM and CSR, despite the imbalance in mutation frequency along the V region.

### V Region Histone Modifications Stabilize AID Substrates and Recruit DNA Repair Proteins to Support SHM

Various histone modifications have been implicated in SHM. Many of these are generally associated with open chromatin and active transcription, while others appear to have more defined roles in actively supporting antibody maturation (Figure 1) (18). A significant histone mark enriched at sites of SHM and CSR is H3K4me3. Transcription elongation factor Spt5 helps to introduce H3K4 tri-methylation through the trans-histone modification pathway (19), alongside the facilitates chromatin transcription (FACT) complex, to support transcription elongation. Spt5 has an additional role as an adapter protein to link AID and RNA polymerase II (20).

H3K4me3, SMARCA4, and FACT complex components are equally important for recruitment of Topoisomerase I (Top1) (21). Top1 typically acts to correct transcription-induced negative supercoiling caused by RNA polymerase II by nicking one strand of the DNA helix, passing the other strand through the break, and re-ligating the nicked end. Reduction of Top1 increases SHM mutagenesis, whereas overexpression of Top1 downregulates SHM. Interestingly, treatment with the Top1 catalytic inhibitor, camptothecin, suppresses SHM. These results indicate that the cleavage activity of Top1 is required for SHM and not its ligation activity (22).

The H3.3 histone variant is another feature associated with SHM and is enriched at the VDJ region in chicken DT40 cells (20). H3.3 appears to be responsible for stabilizing the ssDNA substrate for AID activity. R-loops are often cited as a predominant AID substrate in C regions, although treatment with RNase H to remove these R-loops from the V region of wild-type and H3.3-null DT40 cells identified that loss of these structures does not impede accumulation of AID-induced point mutations. H3.3 may instead be responsible for mediating RNA polymerase II pausing, prolonging exposure of the transcription bubble, and promoting AID targeting (23, 24). Other structures have been proposed to facilitate ssDNA exposure, such as the formation of negative supercoils upon activation of RNA polymerase II transcription. It appears that topoisomerase is unable to repair this topological strain at the same rate that RNA polymerase II progresses (25), and this creates localized denaturation bubbles that are ideal substrates for AID (26). Unfortunately, the mechanism by which H3.3 stabilizes ssDNA substrates remains elusive.

Ubiquitination of proteins is an essential modification to propagate repair of mutated regions in SHM; histones and proliferating cell nuclear antigen (PCNA) are well-known targets. Ubiquitinated (Ub) H2AK119 and H2BK120 are specifically associated with V regions, but not with constant region exons (C_H_) (Figure 1). These histones co-localize with translesion DNA polymerase η, which possesses a ubiquitin-binding domain that binds to mono-ubiquitinated PCNA at lysine 164. DNA polymerase η introduces all the A:T mutations in SHM, but limited mutations in CSR, indicating polymerase η of mismatch repair (MMR) is the dominant repair polymerase only in SHM (27, 28). It is not known whether PCNA is Ub before or after being recruited to V regions (29). Surprisingly, the E3 ubiquitin ligase RNF8 is known to ubiquitinate PCNA, yet has only been shown to support CSR (30–32). It is, therefore, possible that although mono-ubiquitinated PCNA enhances the mutation profile for SHM, its downstream repair effects in CSR are selected against, as large regions of DNA containing these mutations are disposed of following recombination at S regions.

Resolution of mismatched bases is heavily dependent on “corrupted” repair mechanisms. However, there are conflicting reports over the relative involvement of MMR and base excision repair (BER) components. DNA uracil glycosylase (UNG) recognizes and cleaves uracil bases from the genome in BER, while MutSα recognizes mismatched bases and recruits several downstream effectors during antibody diversification, e.g., exonuclease I (Exo1) in MMR (33). MSH6 likely promotes SHM and CSR following recruitment by mono-, di-, and tri-methylated H3K36 through its PWWP motif. Many other proteins involved in DNA damage responses and histone modifications also carry this PWWP motif to promote chromatin interactions (34), including PCNA. Indeed, MMR has been implicated as the principle repair pathway in SHM, following observations that the absence of UNG of the BER pathway has very little impact on the accumulation of A:T mutations, and loss of MSH2, MSH6, and Exo1 lose 80–90% of A:T mutations independently (35). Interestingly, PCNA also interacts with MSH6, which may account for its targeting to appropriate regions during antibody maturation (29, 36). However, it is worth noting that these latter chromatin modifications are not specific to antibody genes and could happen genome-wide. Though it is possible that they acquire added importance by being combined with antibody gene-specific chromatin valencies (37).

One histone modification without a clear role in SHM is H2BS14ph (38). While it serves as a marker for SHM in B1-8 GC B cells, the implications of losing this modification are unknown. H2BS14ph is not present at VJλ1, V_H_, or Sμ in naïve B cells, or B cells 14 days post-activation, but was reproducibly detected at day 10. Consistent with these observations, the only known H2B kinase, Mst1, is present at these sites only at day 10 (38). It is possible that this histone mark is linked to a distinct DSB repair response at V regions around day 10, whereas γH2AX is associated with DSB repair at S regions (38, 39). The strict temporal restriction of the occurrence of this histone mark at day 10 may signify that AID-dependent lesions occur at earlier stages of the GC response, or it may only be required at earlier stages, perhaps for recruitment of downstream proteins (38).

## DNA and RNA Structures Target AID to S Regions for CSR

Class-switch recombination is achieved through the generation of DNA DSBs and subsequent ligation of two distal S regions (12). Transcription alone cannot determine deamination targets for AID, as many genes transcribed in activated B cells are not targeted by AID (40). Instead, S regions encode unusually high densities of the overlapping AID hotspot WGCW sequences that place two WRC motifs in on opposite strands of the dsDNA helix. AID preferentially deaminates the underlined cytosines and, in the event of parallel deaminations, the resulting nicks on each strand (following UNG and APE1 activity) would inevitably produce a DSB. CGC is yet another hotspot, although it rarely appears within S regions. Indeed, WGCW density correlates strongly with CSR efficiency, much better than when WRC alone was considered to predict S region quality (41).

### DNA Secondary Structures Affect Mutation Targeting Preference

Recent work has shown how AID preferentially binds guanine-rich DNA quadruplex structures compared to linear DNA of the same sequence (42). Through dissection of the core quadruplex unit, it was determined that AID binds to the adjacent ssDNA strands at a stoichiometry of AID_2_/DNA (42). It requires a binding site of at least five nucleotides (42). By studying the distance of the deoxycytidine (dC) in hotspot (AGCT) and cold spot (TTCT) motifs from the quadruplex, it became clear that peak deamination occurs when dC is at third position and is independent of the sequence, suggesting that the quadruplex structure overrides sequence motif preferences (42). However, as the dC is shifted further from the quadruplex, this preference for AGCT is approximately double that of the TTCT, recapitulating hotspot preference seen in a multitude of *in vitro* and *in vivo* assays (43, 44). Interestingly, in S regions, a dC is often present at precisely the third position from the G-repeat (42). This binding preference is also observed in RNA quadruplexes (42). Accordingly, AID has two DNA-binding faces: the substrate binding channel and the “assistant patch” (42). In such a model, the assistant patch enhances AID affinity for the substrate, and increases its deamination activity. This bifurcate binding structure is unique to AID, and is not seen in AID homologs, explaining why this bifurcate binding phenomenon and cooperativity is not observed in APOBEC3A or APOBEC3G (42).

This preferential binding to quadruplex DNA has previously been observed, whereby AID targets proto-oncogenes to introduce translocations at c-MYC and BCL-6, among others (40). Lymphomas in which these proto-oncogenes are unstable derive from GC B cells. For example, a hallmark of Burkitt’s lymphoma is c-MYC recombination with S regions, promoting deregulated expression of this crucial gene (45). In addition, c-MYC, PAX5, and BCL-6 translocations are associated with progression from follicular lymphoma to the more aggressive diffuse large B cell lymphoma (46), and PAX5/IgH translocations have been identified in a subset of non-Hodgkin’s lymphomas (47). This genomic instability does not correlate with WRC sequence, but instead correlates with G-rich regions (40). Furthermore, this G-richness does not characterize translocation breakpoints in AID-null B and T cell malignancies (40). Most translocations associated with leukemias in AID-null cells results from a mechanism that is independent of G-rich content, yet the data suggest that in GC B cells in which AID is highly expressed, AID preferentially targets transcribed G-rich regions, and therefore, its stringent targeting to the Ig region is essential to maintain genomic stability.

### RNA Secondary Structures Also Contributes to Mutation Targeting

Transcription through S regions has been associated with AID targeting to the IgH locus through the formation of R-loops and the interaction between AID and RNA polymerase II (24). While germline transcripts through I_H_–S_H_–C_H_ regions have been implicated in antibody diversification, their mechanistic function has only recently been demonstrated. AID binds directly to sense germline transcripts as well as to telomere RNA (48). These transcripts are also G-rich and form G-quadruplex structures. Ablating the G-quadruplex structure through G-to-C mutations, or inhibiting the splicing machinery that supports formation of these secondary structures, disrupts AID interaction with the transcripts and concomitantly reduces CSR (48). Amino acids 130–138 in AID show homology to the RNA-binding domain of RHAU, a known binder of G-quadruplex RNAs. Mutations in this binding region also perturb AID:RNA-binding capacity, consistent with hyper-IgM patients possessing a G133V mutation (48). Whether this mutation impedes AID:DNA binding is unknown, though it may be involved in the transfer of AID from its RNA guide to the DNA substrate. This also suggests that some RNA splicing proteins such as PTBP2 and CTNNBL1 may also play an indirect role in CSR by shaping the ncRNA architecture (48–50).

Although this provides a detailed explanation of the mechanism behind AID targeting to S regions for CSR, it fails to explain AID targeting to V regions. More research is needed to determine if this RNA-binding capability is indeed distinct for CSR, as it may be responsible for deviance in processes downstream of AID-induced mutation in SHM and CSR. As previous reports have shown that the C-terminal amino acids 189–198 are vital only for CSR (51, 52), it is unlikely that the RNA-binding region explains this whole process.

Studies suggest that the RNA exosome complex is recruited to S regions in an AID-dependent manner, and makes the transcribed strand accessible to AID deaminations by degrading the complementary-bound nascent RNA strand (53). Knockdown of the RNA exosome reduces CSR by 30–50% compared to controls. The RNA exosome also promotes quality control on the antisense transcription of ncRNA from TSSs, which have the ability to both recruit AID and generate ssDNA substrates for its catalytic activity (54). By degrading superfluous antisense RNAs, which increases the formation of RNA:DNA hybrid structures and heighten risk of premature transcription termination and genomic instability, the RNA exosome protects genomic integrity (54). In addition, the RNA exosome appears to control a long ncRNA expressed from a divergent enhancer element, which directly regulates the 3’RR of the IgH locus by enhancing the looping activity known to promote CSR activity (55, 56) (Figure 1). Unexpectedly, although histone acetylation and deposition of H3K4me3 coincides with B cell development stages along the *Igh* locus, major epigenetic alternations have not been detected at the 3’RR upon splenic B cell activation (57, 58).

### miRNA Control of Antibody Production by Regulation of SHM and CSR

It has been well-documented that miRNAs can regulate SHM and CSR in B cells, chiefly through modulating AID and Blimp-1 expression (56, 59–62). miRNAs such as miR-155, miR-181b, and miR-361 can silence AID expression (59, 61, 63, 64), whereas miR-30a and miR-125b can silence Blimp-1 expression (65–67), which is required for plasma cell differentiation and antibody production. These miRNAs bind to evolutionary conserved target sites in the 3′UTR of *Aicda* and *Prdm1* mRNAs. More recently, histone deacetylase inhibitors have been reported to repress the expression of AID and Blimp-1 by upregulation of these miRNAs (68).

In particular, the more prominent role of miR-155 in regulating activated B-cells and the GC response is becoming more established. MiR-155 is directly repressed by BCL-6, the master regulator of GC formation, which is upregulated in the dark zone, repressing genes involved in cell cycle arrest, DNA damage response and plasma cell differentiation and thus allowing SHM to take place. miR-155 deficiency in B-cells has been shown to decrease the number of IgG1^+^ plasma cells and memory B cells and abolish the production of high affinity IgG1^+^ antibodies indicating that miR-155 plays a key role in affinity maturation and CSR. More recently, miR-155 has been reported to be involved in the survival of positively selected GC B-cells (69–73).

What is now beginning to emerge, however, is the notion that miRNA can be transferred from one immune cell to another through understudied “epigenetic shuttles” called exosomes that can transport RNA and protein factors (74). Exosome “shuttling” of miRNAs and antigen between B and T cells occurs following construction of the immune synapse (75–77). This may indirectly support CSR by potentiating a feedback loop between T helper cells and activated B cells. B cells persistently stimulate T helper cells to secrete cytokines that promote CSR, such as TGFβ1, IL-2, and IL-4 (78). 12% of B cell-internalized antigen is spared destruction and is instead secreted on exosomes that are received by the bound T helper cells to encourage cytokine production (79). A specific role for miRNAs in directing this targeted approach toward antibody maturation has yet to be elucidated and further research in the regulatory potential of this process is required.

### Histone Modifications Decorate the Donor and Recipient S Regions to Recruit AID in CSR

Specific cytokine stimuli act on activated B cells to drive recombination between donor and desired recipient S regions to select for a particular Ig isotype. The Sμ region is always primed for class switching as histone modifications that are generally associated with an open chromatin state (including H2BK5ac, H3K9ac, H3K14ac, H3K27ac, H4K8ac, H3K4me3, and H3K36me3) are enriched at this site prior to antigen-engagement. As such, Iμ–Sμ–Cμ transcripts are also constitutively expressed (5, 80–86). The remainder of the chromatin modifications could be broadly categorized into two general pools, targeting modifications upstream of AID recruitment and downstream modifications mostly associated with the general DNA damage response (Figure 1). Indeed, acetylated H3 and H4 fall broadly within these two categories as H3ac correlates with germline transcription in unstimulated splenic B cells, while H4 acetylation is observed following B cell activation, likely in response to AID-induced DSBs (80). This is observed in the 1. B4.B6 B cell line. These B cells undergo CSR to γ3 upon treatment with LPS + CD40, and CSR to γ1 and ε1 following treatment with LPS + CD40 + IL-4. Following LPS + CD40 treatment, γ3 GLTs are induced, while γ1 and ε1 GLTs are repressed. Correspondingly, H4ac levels at Sμ, Iγ3, and Sγ3 are increased, whereas S regions and promoters for γ1 and ε1 loci are marginally affected. The reciprocal is observed upon LPS + CD40 + IL-4 treatment for GLT expression and H4ac. This suggests that regions of chromatin are specifically remodeled to identify the S region for AID mutation (80).

Permissive transcriptional histone marks are abundant in S regions, including H3K4me1/2/3. NHEJ-compliant protein PTIP typically facilitates distribution of these marks through its interaction with MLL3/MLL4 to support DNA repair and transcription. Unexpectedly, the interaction between PTIP and MLL3/MLL4 is dispensable for *Igh* germline transcription, and is mostly responsible for H3K4me1/2 production. Recently, a sub-complex made up of PTIP-PA1 appears to promote H3K4me3 formation through other unidentified histone methyltransferases. The function of this complex facilitates the transcription preceding AID deaminations, promoting CSR to IgG isotypes, and appears to have very little influence on DNA repair (87, 88). Nevertheless, MLL4 is important for maintaining effective CSR; it is frequently mutated in diffuse large B cell lymphoma and follicular lymphoma (89), and hypogammaglobulinaemia is common in the heritable Kabuki syndrome, often attributed to MLL4 mutations (90).

Specific chromatin modifications have been implicated as markers for the donor and recipient, and thus as possible recruiters of AID and/or other components of the CSR machinery. Tri-methylation of H3K4 is facilitated by the FACT complex. Knockdown of FACT components SSRP1 and SPT16 in the CH12 B cell lines results in a significant decrease in IgA switching (85) and corresponds with an overall decrease in H3 methylation in the Sμ region and a specific reduction of H3K4me3 in the Sα region. The components acting downstream of the H3K4me3 marker that lead to CSR remain elusive, although DNA cleavage assays have shown that breaks in the Sμ and Sα regions are significantly reduced in SSRP1 and SPT16 knockdown cells (85, 91).

H3K4me3, SMARCA4, and FACT help mediate CSR through recruitment of Top1, as they do for SHM (21, 85). Reduced levels of Top1 renders it unable to keep pace with RNA polymerase II, accumulating negative DNA supercoiling at the rear. Repeat sequences and palindrome-rich regions are prone to this non-B DNA structure and are prevalent in S regions (92). In addition, there is an interesting relationship between AID expression and Top1 levels. AID overexpression coincides with abated Top1 mRNA translation, the mechanisms of which have not been thoroughly explored (92).

AID has recently been shown to interact with Suv4-20H H4K20me methyltransferases, though whether this is a direct interaction, or mediated through other proteins or RNA structures, is not known (93). Without AID, Suv4-20H is not recruited to S regions, and the level of H4K20me3 is reduced at these sites (93). Concordantly, *Suv4-20h* double-null mice are defective in CSR (94). It has been proposed that H4K16ac and Suv4-20H-mediated H4K20me3 play antagonistic roles in RNA pol II pausing. H4K16ac promotes release from pausing, while H4K20me3 prolongs RNA pol II pausing (95). AID-induced mutations are long-understood to be reliant on RNA pol II pausing, so it is possible that AID reinforces this pausing step through Suv4-H20 recruitment (24). However, this has not been confirmed.

Histones H3K9me3 and H3K9ac decorate S regions that undergo recombination (5, 84). These modifications are dependent on cytokine stimulation but are independent of AID expression. It has, therefore, been suggested that the two histone marks precede AID-induced mutations and recombination and perhaps even function in the recruitment of AID to the appropriate sites (84). H3K9me3 has been shown to be essential for general DSB repair through its direct interaction with the lysine acetyltransferase Kat5 and loss of H3K9me3 results in defective DSB repair (96, 97). The link between DSB recognition and H3K9 methylation is currently unknown; however, it is understood that it participates in NHEJ, indicative of a role in CSR, but not SHM or the preceding V(D)J recombination. H3K9 is methylated by its methyltransferase, Suv39h1, which exists in a complex with kap-1 and HP1. HP1 possesses a chromodomain, which binds to the newly tri-methylated histone and retains the complex at the S region site.

There is specific evidence supporting a role for H3K9me in CSR. The kap-1 and HP1 complex functions as the structure that tethers AID to Sμ (6). Similar to the G-rich quadruplexes mentioned previously, the binding of AID to kap-1 is not reliant on its C-terminal domain and, as such, it is unlikely that this association explains the requisite of the C-terminus for CSR (6).

H3K9ac phosphorylated at serine 10 (S10ph) is another histone modification that has been implicated as a marker of donor and recipient S regions. This mark has been found to be enriched at the donor Sμ region and, after B cell activation, in the cytokine-selected recipient S region (98). 14-3-3 adapters interact directly with H3S10ph and the affinity of this interaction is increased with the addition of an acetyl group on lysine 9 of the same histone (99, 100). ChIP assays have shown that, upon lipopolysaccharide stimulation, 14-3-3 is recruited specifically to the S regions enriched in H3K9acS10ph (98). Upregulation of the 14-3-3 complex coincides with CSR. The complex directly binds AID and associates specifically with 5′-AGCT-3′ motifs that occur frequently in S regions and are particularly common within the V region. Reduced 14-3-3 activity correlates with a decrease in AID at active S regions (101). This implies that 14-3-3 is an important factor for recruiting AID and associated proteins to recombination sites for CSR. It seems H3K9acS10ph recruits and/or stabilizes 14-3-3, which in turn recruits AID to the appropriate S region.

### Chromatin Modifications Recruit DSB Repair Proteins in CSR

**Figure 2:**
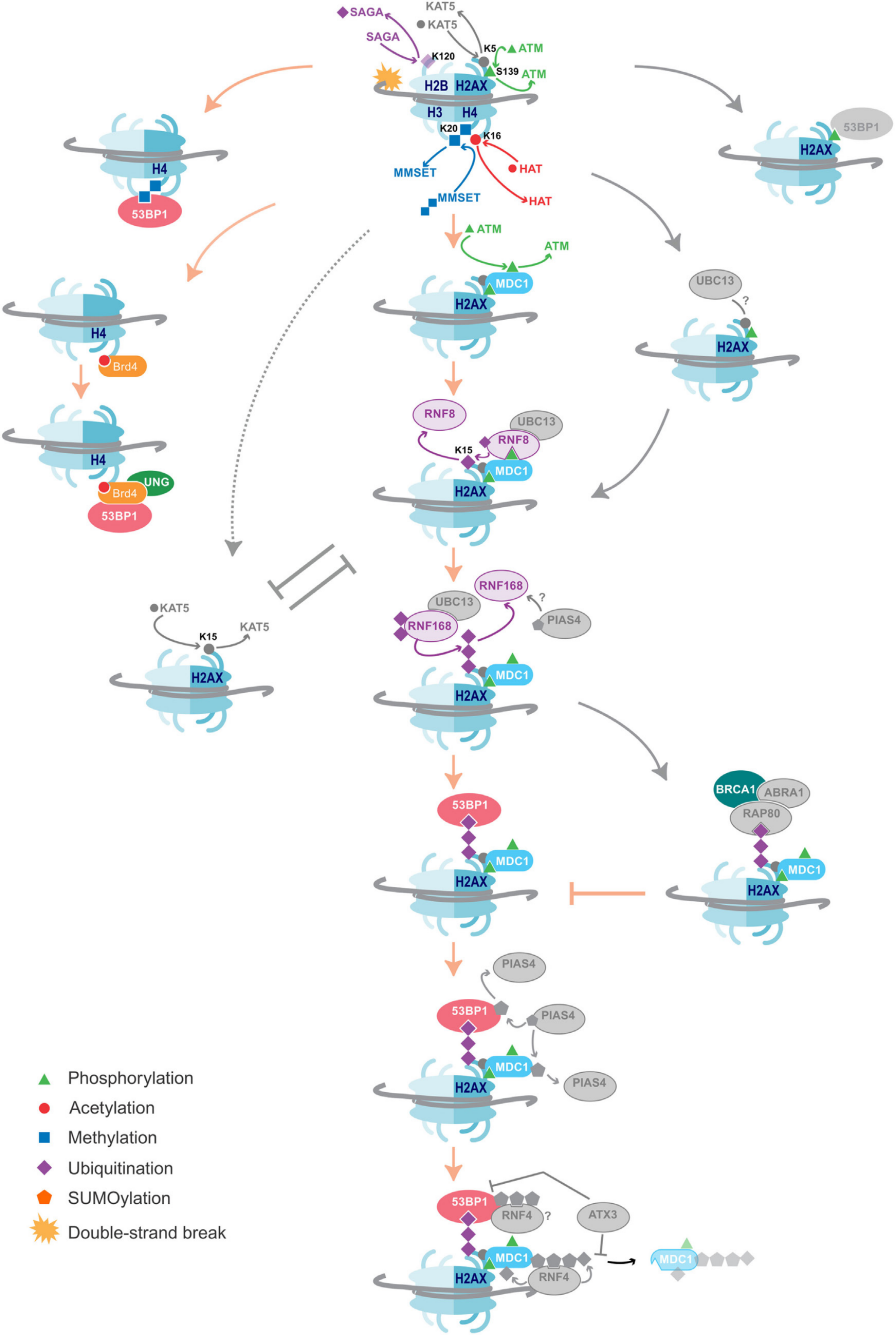
DNA damage repair pathways dictate class-switch recombination (CSR) efficiency. An overview of the histone modifications, and the writers and readers associated with them that are essential for CSR, as well as suggestions of additional likely factors. Color = confirmed in NHEJ and CSR, Gray = DNA damage repair factors not yet shown to affect CSR. The key proteins and histone modifications that have been shown to be essential for resolving the DNA double-strand breaks in CSR are summarized. A common theme is the recruitment of 53BP1, which is essential for efficient repair and isotype switching. Importantly, these repair pathways also function in NHEJ. Additional DNA damage repair proteins and histone modifications that have not yet been shown to play a role in CSR are indicated in gray. Some proteins and histone marks involved in other repair pathways, such as homologous recombination (HR), are also indicated in the figure. As these pathways inhibit the NHEJ pathways, they may provide negative control of CSR. Indeed, knockdown of BRCA1 has been shown to increase isotype switching efficiency.

Chromatin markers participate in the recruitment of the required repair proteins. 53BP1 is one protein confirmed to hold an essential role in DSB repair and promotes NHEJ for CSR by bridging the broken ends (102–108). Recruitment of 53BP1 to DSBs is dependent on various chromatin modification pathways (Figure 2). It is a bivalent chromatin reader and interacts directly with the histone marker H4K20me2 through its tudor domain, which recognizes methylated histones (109, 110). Independent of its role in S region DSB repair, 53BP1 exerts a secondary influence on CSR by enforcing the temporal separation of Sμ and Sγ breaks and ensures that subsequent ligation of the broken ends results in a deletion event (111). It does this by orchestrating the preferential breaking of the upstream switch region Sμ. *53bp1^−/−^* B cells lose the ability to ensure Sμ breaks first, which introduces inversional rearrangements that negatively impact CSR efficiency (111). 53BP1 recognizes H4K20me1 *in vitro*, but it is its specific recognition of di- and tri-methylated H4K20 made accessible to 53BP1 during the DNA damage response that may regulate break order in CSR (109).

Depleting cells of SUV4-20H1/H2, the predominant methyltransferases producing H4K20me2, slows the rate of 53BP1 accumulation at break sites and reduces CSR efficiency by 50%; however, the absence of the H4K20me2 mark has no impact on S region break order (112). This may be due to the activity of another H4K20me1 methyltransferase called MMSET that had not been considered by Rocha et al. Indeed, loss of MMSET hampers H4K20me2 enrichment, significantly reduces 53BP1 binding, and leads to inefficient CSR (113). MMSET specifically methylates H4K20me1 and H4K20me1/2/3 are all locally increased at DSBs (Figure 2) (114). This may explain why the loss of SUV4-20H1/H2 only slows 53BP1 recruitment and does not completely abolish it. Additionally, MMSET, and not SUV4-20H1/H2, is uniquely overexpressed in GC B cells, possibly ascribing MMSET as the dominant methyltransferase in antibody diversification (115). MMSET is activated and recruited after ATM-mediated phosphorylation during typical DNA repair, allowing it to complex with MDC1. MDC1 binds γH2AX, a mark only introduced once DNA repair signaling has been initiated (114). MDC1 is important for enlisting ubiquitin ligases RNF8 and RNF168, which lay a polyubiquitin motif also recognized by 53BP1 (31) (Figure 2).

Unfortunately, it is impossible to study the impact of H4K20me1 knockdown on CSR efficiency as it is a global histone mark implicit in proliferation and cellular viability (116); however, introducing a single point mutation in the 53BP1 Tudor domain, preventing it from recognizing H4K20me1/2, disrupts S region break order. Taken together with the aforementioned findings that the absence of H4K20me1 has no impact on S break order, these suggest that the dimethyl mark is dispensable and that it is the H4K20me1 mark that determines break order (111).

Ubiquitin ligases are proving to be pervasive in DNA repair, including CSR. RNF168 monoubiquitinates H2A on 13 and 15 lysine residues (117, 118). Knockdown of either RNF8 or RNF168 results in a decrease in 53BP1 accumulation at AID-induced DNA breaks and a corresponding reduction in CSR is observed (31). Furthermore, expression of a ubiquitin-H2AX fusion protein can rescue 53BP1 recruitment to DSBs in RNF8- or RNF168-deficient cells (119). In the absence of DNA damage, Polycomb group protein L3MBTL1 and demethylase JMJD2A mask H4K20me2. RNF8 and RNF168 are responsible for ubiquitinating these proteins, removing them from the damage site to expose H4K20me2 (120, 121). This secondary role of the ubiquitinases has not been explored in the context of CSR. The bivalent binding by 53BP1 to H4K20me2 and ubiquitin marks could serve to correctly orientate 53BP1 for it to bridge across a DSB. Delayed accumulation of these histones marks might prevent 53BP1 from orientating correctly, which would thus lead to increased CSR inversion events. Methyl and ubiquitin modifications appear to have different influences on 53BP1. H4K20me2 more likely serves as a signal to recruit 53BP1 to the DSB, while ubiquitination H2A/H2AX serves as an anchor to 53BP1, maintaining it at the site of the DSB, such that 53BP1 can bridge the gap between donor and recipient S regions for isotype switching.

Upstream in the signaling cascade, a deubiquitination event also promotes DNA repair and CSR. Ubiquitination of H2BK120 is associated with an open chromatin and interferes with chromatin compaction. DSB repair cannot occur until histone H2BK120ub is deubiquitinated to allow access to NHEJ factors (122). A genome-wide loss-of-function RNAi screen identified several components of the SAGA deubiquitinase module required for CSR and DSB repair, including Usp22, Eny2, and Atxn7. Knockdown of any of these components using shRNAs or CRISPR/Cas9 reduces CSR (123). Knockdown did not impair AID function indicating that the defect lies somewhere downstream. Interestingly, Eny2 knockdown also interferes with ATM and/or DNAPK activity, and thus indirectly limits γH2AX formation, which further reduces CSR (123, 124).

BET family member Brd4 interacts with acetylated histones *via* its two bromodomains (125). Studies have shown that, upon induction of AID, occupancy of Brd4 at Sμ and Sα regions increases. ChIP and immunoprecipitation assays have confirmed an interaction between Brd4, the modified histones H4 and γH2AX, as well as between Brd4, 53BP1, and UNG (126). Treatment with the Brd4 inhibitor JQ1 or siBrd4 knockdown significantly reduces CSR frequency. The levels of both 53BP1 and UNG are reduced, without affecting the levels of H4ac. Brd4 is, therefore, thought to function as a chromatin-bound platform that recruits 53BP1 and UNG to DSBs (126) (Figure 2). Finally, the chromatin remodeling complex INO80 has also been implicated as a regulator of CSR (127). Knockdown of INO80 in various mammalian cell lines has been shown to inhibit 53BP1 accumulation at DSBs (128). More recently, MEFs from *mino80* knockout mice contradict this observation. Rather, INO80 is suggested to participate early on during DSB repair, where it first binds γH2AX, and then exposes H4K20me2 for 53BP1 recruitment. Paradoxically, INO80 is involved in 5′–3′ DNA end resection to support repair by homologous recombination (HR) (129). How it then functions to support NHEJ in CSR is yet another mystery.

### Potential Role for Other Repair Proteins in CSR

The histone modifications and DNA damage repair proteins important for CSR (Figure 2) have parallel roles in NHEJ. This is particularly interesting because a multitude of proteins, modifiers, and readers involved in NHEJ have not yet been implicated in CSR. These include the E2 ubiquitin-conjugating enzyme Ubc13, which functions in complex with RNF8 and RNF168. Ubc13 and its γH2AX independent recruitment through the Kat5 complex (130–133) are potential important factors in CSR. Indeed, H2AX-deficient mice experience reduced CSR (39), while a link with SHM fails to be seen (38). A SUMOylation pathway, initiated by PIAS4 (134, 135) and further expanded by STUbL RNF4 (136), provides a potential role of SUMO-ubiquitin cross talk in CSR. The importance of this pathway in general DNA damage repair is exemplified by Ataxin-3, which counteracts the RNF4-mediated ubiquitination. As a result, Ataxin-3 promotes prolonged retention of MDC1, resulting in reduced recruitment of 53BP1 and BRCA1 (112).

The possible impact of NHEJ regulatory factors specifically should be considered on antibody diversification. The recently discovered tudor interacting repair regulator (TIRR) stabilizes 53BP1 in the nuclear fraction, but blocks NHEJ-directed repair by binding the tudor domain and guarding against H4K20me2 binding upon DNA damage (137). As such, it may act to hinder CSR when either over- or under-expressed, and would determine whether the turnover rate or instability of 5BP1 will compensate for more favorable H4K20me2 binding. Equally, the contribution of RIF1 in suppressing DNA resection for NHEJ and separating the 53BP1–TIRR complex may similarly serve to deregulate CSR at differential expression levels (138). The HR pathway also represents a potential research avenue as it may provide inhibitory effects on CSR efficiency. Knockdown of BRCA1, a key HR factor, has been shown to increase isotype switching (139). Similar effects are observed from downregulation of other inhibitory modifications, such as the H2AXK15ac by the Kat5 complex, which inhibits the RNF8 mediated ubiquitination of H2AXK15 (140).

## Influence of AID and TET Activity on the DNA Methylome During B Cell Development

### Role of AID in DNA Demethylation *via* Deamination

Aside from its mutagenic activity, AID has been associated with coordinating DNA demethylation during zebrafish development (141), stem cell reprogramming (142), and primordial germ cell formation (143). The combined results of these studies support the notion that AID could function as a genome-wide epigenetic regulator by deaminating 5-methylcytosine (5mC) to 5-methyluracil; thereby replacing a 5mC base with an unmethylated C or a thymine (T) *via* BER. GC B cells have more heterogeneous DNA methylation patterns than naïve B cells (4), and this has established a potential role for AID during this maturation step.

Several studies have debated whether AID is responsible for DNA demethylation or activated gene expression in B cells (144). The methylation status of CpG motifs at VDλ1 is unchanged between naïve and day 14 GC B cells (38), AID does not induce demethylation at either Sμ or Cμ (93), and 5mC is a poor substrate for AID, although it does not prevent its activity on neighboring cytosines (145, 146). In contrast, fewer studies have found that DNA demethylation events can be attributed to AID. CpGs have been observed to have increased methylation pattern variation in wild-type tissues, compared to AID-null tissues. Interestingly, 90% of the methylome alterations seen in naïve to GC transition were lost in AID-null mice (147). SHM targets are also suggested to be enriched with AID-dependent hypomethylation, and the significant reduction of both demethylation and SHM *ex vivo* (such as in the contribution by Fritz et al.) is due to these two events being coupled *in vivo* (147). Furthermore, a recent study suggested that cytosine demethylation is over-represented in WRCG/CGYW motifs in follicular lymphomas, which overlays the WRC AID hotspot motif and the methylated CpG dinucleotide. This contrasts SHM of Ig genes whereby cytosine demethylation is under-represented at WRCG/CGYW motifs. Thus, this mutational process appears distinct from conventional SHM, and is solely applied to the CpG methylation/demethylation process (148).

### Role of TET Protein in DNA Demethylation *via* Hydroxylation

As the involvement of AID in DNA demethylation remains to be fully established, the regulation of DNA methylation by another family of proteins is now being explored. Ten-eleven translocases (TET1, TET2, and TET3) oxidize 5mC to 5-hydroxymethylcytosine (5hmC) and further oxidizes 5hmC to 5-formylcytosine (5fC) and 5-carboxycytosine (5caC) (149, 150). TET proteins predominantly support demethylation *via* dilution through successive rounds of replication (149). Nevertheless, it is possible that TET enzymes support active (replication-independent) demethylation. TET enzymes often accompany transcription-associated H3K36me3 histone modifications, and possibly RNA polymerase II, depositing 5hmC and generating a more accessible DNA substrate for subsequent cycles of transcription (151). TET enzymes are involved in iterative rounds of 5mC oxidation to 5fC and 5caC (150). Demethylation could then be achieved either (i) indirectly *via* thymidine DNA glycosylase which recognizes and excises 5fC an 5caC (152) or (ii) directly by yet unidentified decarboxylases (153, 154).

TET proteins appear to be important for programming B cell methylation throughout development. 5hmC is enriched in lineage-specific transcription factors, such as *Bcl6, EBF1*, and *IRF4*, which are important for GC transition (155, 156). The methylation status of follicular B cells from conditional *Tet2^−^/Tet3^−^* double knockout mice were partially hypermethylated when compared to wild-type cohorts. Single knockout mice failed to show such noticeable effects on methylation levels (155). Of the sequences that are specifically demethylated in wild-type B cells during differentiation into GC B cells, 95% are prevented in *Tet2^−^/Tet3^−^* mice, providing some evidence that TET proteins may be responsible for most DNA demethylation that occurs at this stage (155). In addition, the Igκ locus is known to undergo DNA demethylation during antibody diversification, and this demethylation step is not observed in *Tet2^−^/Tet3^−^* knockout mice (155).

### Cooperation between AID and TET Proteins during Epigenomic Regulation

In addition to regulating B cell development, TET proteins are essential tumor suppressors in B cells. Of all patients diagnosed with diffuse large B cell lymphoma, 5.7% carry a Tet2 deletion or loss-of-function mutation (157). In mouse genetic studies, Tet1-deficient B cell progenitors developed B cells lymphomas (158); in analogous human studies, the Tet1 promoter was found to be hypermethylated with concomitant reduction in Tet1 expression in patients with non-Hodgkin lymphoma (158). In additional studies, mice with a combined Tet2- and Tet3-deficiency in developing B cells developed B cell lymphoma and succumbed to disease within 5–6 months of age, much earlier than the 15–20 months observed in Tet1/Tet2-deficient mice (159, 160).

It has been proposed that the product of TET protein-dependent 5mC oxidation may be a target for AID. Few studies have addressed the cooperative activities of TET proteins and AID. Some have concluded that it is unlikely that AID deaminates 5mC or 5hmC: 5mC is deaminated only at 10% the rate of cytosine due to the steric hindrance of the methyl group (146). 5hmC is an even poorer substrate for AID (161). Deamination of 5hmC *in vitro* has not been observed, and *in vivo* studies overexpressing AID have also failed to generate 5hmU (146). As 5hmU is not yet detected in genomic DNA, AID targeting 5hmC as a target for deamination was claimed unlikely, and further supports a role for TET enzymes in B cell developmental demethylation (146). On the contrary, a study in 2011 found that AID quite significantly promotes 5hmC demethylation in HEK293 cells and in mouse brain. While overexpression of AID had little effect on the demethylation of a strand of 5mC-GFP DNA, it led to a significant decrease of 5hmC levels induced by TET1 and significant increase of 5hmU (162). This is significant because there was no detectable endogenous 5hmU in HEK293 cells. Additionally, the pattern of 5hmC demethylation events were broadly distributed along the 5mC-GFP DNA, 5hmC was also selectively demethylated at WCR “hotspot” motifs, and demethylation showed strand bias in the same manner as AID deamination (162). Taken together, this could indicate that AID and TET may act in tandem to promote DNA demethylation. Whether this is replicated in the context of antibody diversification is yet to be seen.

## Epigenomic Role of Immune Diversification in Disease Development

AID defects are associated with hyper-IgM syndrome, causing severe immunodeficiency (163). The epigenetic effect of AID on health, however, particularly lymphomas, is poorly understood. DNA methylation’s role in gene silencing makes it essential in regulating normal development, with epigenetic mutations allowing cells to grow and reproduce uncontrollably, leading to tumorigenesis. DNA methylation can have malignant effects through two main alterations: hypermethylation of tumor suppressor genes and hypomethylation of oncogenes. It has come to light in recent years that such mutations are a common cause of B cell lymphomas, with hypomethylation in GCB-derived lymphomas correlating with AID expression (164). Off-target effects of AID are also seen in non-B cell cancers, for example, T cell malignancy (86), and also in non-lymphatic cancers, such as stomach cancer (165), lung cancer (166), breast cancer (167), and liver cancer (168).

From this observation, it could be hypothesized that ectopic AID expression plays a critical role in lymphomagenesis. Increased epigenetic heterogeneity in lymphomas reflects diverse tumor cell populations, which increases risk of resistance to cytotoxic drugs (164). Understanding AID, and its role in lymphomas, could provide guidance in the development of new epigenetic drugs. Currently the main epigenetic cancer therapy drugs are azacytidine and decitabine which function as DNA methyltransferase inhibitors, combating DNA hypermethylation. These drugs have shown substantial potency in reactivating epigenetically silenced tumor suppressor genes *in vitro* (169). Reducing levels of AID could be used in a similar way against hypomethylation or the resistance caused by epigenetic heterogeneity in lymphomas. The protein HSP90 is important in the protection of AID from proteasomal degradation, with inhibition by the drug 17-AAG, leading to polyubiquitination and degradation of AID (170). 17-AAG is currently in clinical trials for the treatment of other cancer types, due to its role in inhibiting the degradation of proteins involved in tumor cell proliferation and survival (171). The above observations suggest a possibility of using 17-AAG in the treatment of hypomethylated lymphomas (Figure 1). In a recent study 17-DMAG, a derivative of 17-AAG, has been found to reduce CSR and SHM in mice, while B-cell survival and proliferation remain unaffected (172).

## Concluding Remarks

The epigenome is made up of several critical components that must work together to promote antibody maturation and diversification in B cells. This is an intricate process; each component simultaneously functions both independently and dependently on the others, and disruption at any step can have catastrophic downstream affects. For example, histone modifications relax the chromatin, allowing for AID transcription. Simultaneously, multiple different ncRNAs regulate transcription and target AID to mutate Ig region genes. Next, different histone modifications recruit DNA repair proteins which then multiple different ncRNAs target. The entire process is further complicated depending on *which* histone modifications are used and *which* ncRNAs are present whether a B cell is returned to the *status quo*, undergoes CSR, or undergoes SHM. It is a tremendously complicated process and abrogation at any step can result in various forms of cancer and/or immunodeficiencies. Despite advancements of our knowledge of this field, several important questions remain unanswered. These include the mechanisms controlling AID transcription and the mechanisms that direct AID to target neutral, CSR, or SHM region genes. Furthermore, we have yet to determine how ssDNA is stabilized for AID activity.

## Author Contributions

RC conceived the theme/direction. ECS, RBM, MJD, RL, and RC researched and wrote the manuscript. ECS and RBM designed the figures.

## Conflict of Interest Statement

The authors declare that the research was conducted in the absence of any commercial or financial relationships that could be construed as a potential conflict of interest.
